# An Integrated Approach to Current Trends in Organic Food in the EU

**DOI:** 10.3390/foods8050144

**Published:** 2019-04-26

**Authors:** Ionel Bostan, Mihaela Onofrei, Anca Florentina Gavriluţă (Vatamanu), Carmen Toderașcu, Cristina Mihaela Lazăr

**Affiliations:** 1 Faculty of Law and Administrative Sciences, Stefan cel Mare University, 13 Universităţii, 720229 Suceava, Romania; 2 Faculty of Economics and Business Administration, Al. I. Cuza University, 11 Carol I, 700506 Iaşi, Romania; onofrei@uaic.ro (M.O.); anca.vatamanu@mail.uaic.ro (A.F.G.(V.)); carmen.sandu@uaic.ro (C.T.); 3 Faculty of Economic Sciences, Ovidius University, 58 Ion Vodă, 900527 Constanta, Romania; cristina.lazar@365.univ-ovidius.ro or lazarcristinam@yahoo.com

**Keywords:** organic food, human health, food security, soil protection, Codex Alimentarius Guidelines

## Abstract

The objective of this paper is to summarize an extensive bibliographical search by presenting the retrospective of EU organic food from the point of regulation, policy framework, trends and challenges. We also make a critical review of existing scientific evidence regarding European trends in organic food consumption and production and we identify research gaps for future comprehensive assessments of the policy and legal framework. This review has indicated the importance of the two pillars, namely regulation and policy, highlighting not only the need for solid restrictions regarding organic food production, however also the need to support food safety and consumer confidence in the growing organic food market.

## 1. Introduction

Conceptually, the organic food is a result of the organic philosophy practices and principles [1,2,3]. Some authors highlight the subject from the “biological” or “natural production” point of view [4,5] while others emphasize the relationship with “green concepts” and “environmental friendliness” [3,6] and another category is linked to the idea of implication of artificial chemicals in organic production [7,8,9]. An interesting point of view is found in the work of Vindigni et al. [10], which argued that the term organic often takes into consideration a “process claim” and not a “product claim”. On another side of the spectrum, Velten et al. [11] also claim that sustainable agriculture should impact positive renewable resources and rural development by integrating appropriate natural biological cycles and controls.

Even if in some cases non-organic production usually has higher-yielding cultivars with disease and pest resistance [12], due to the application of inorganic fertilizers and their negative influence on soil [13,14,15,16,17,18], environmental sustainability [19,20,21,22] and human health [23,24,25], the contemporary period started to show a reorientation on the global stage to the idea of eco-friendly and organic food. Since soil erosion is one of the most serious threats facing world food production [26,27], in the absence of some mechanisms and standards to support organic producers, we could talk not only about various consequence on agricultural systems, however the issues related to sustainable development and environmental protection will also be deepened.

Based on the EU framework and standards, organic products are foods grown in a sustainable way, with genetically modified organisms and the use of conventional pesticides, synthetic fertilizers, antibiotics or other substances not being allowed. Organic products consume up to 40% less energy than other products simply because pesticides and fertilizers are not used. The rationale for excluding all the substances mentioned above also supports the requirement that animal welfare conditions are to be respected by raising them in a free range and open-air environment [28,29]. Hence, since social, economic and environmental benefits of organic agriculture increase not only EU interest however also global interest, the main concern is whether the regulation of organic farming of the EU creates a solid framework regarding all the requirements in terms of food labelled as “environmentally-friendly”, “green” and how the consumption of this type of food supports human health improvement and indirectly positively affects environment sustainability. In order to respect organic principles, it is necessary for farmers to consolidate an efficient management, then the final output can consist of better promotion of soil fertility, biodiversity conservation and improvement of life quality.

The theoretical frameworks regarding the exploration of organic consumption highlight that in developed countries, the demand for organic products continues to substantially increase each year. It is supposed that ecological farming is intended to produce healthier food and according to Baudry et al. [30], there is a strong relationship between the level of organic food preferences and health outcomes. The EU organic market increased in 2016 by 11.4% (which means that it nearly reached 33.5 billion euros). According to the European Section of The Organic World [31], it is noticed that in Western European countries, between 2015 and 2016, the market experienced a growth of 5.4 percent and in Eastern Europe, sales recorded a growth rate of 8.8 percent, which means constant requirements and improvements in terms of food security. Climate change and food security become an issue of international accountability and involve concerns regarding environment protection, healthy life, quality in food production and sustainable agriculture. In line with the last point of view, some papers stressed the idea that food security could be improved by focusing on orphan crops, food security plans at the city and municipal level and integrated management [32,33,34], while others pay attention to the idea of soil organic carbon as a strategy to enhance food security [35], agricultural biodiversity [36] or the avoidance of chemical control [37].

Having worked toward the obligation to feed a growing world population, the objective of sustainability lies at the heart of organic production, being one of the most important elements that determine the acceptability of a common framework in terms of rules, production practices and inputs used. Public inspection and certification or the accreditation of private inspection and certification bodies imply a solid legal framework that is capable to ensure fair competition among producers and sustained international trade. Strong regulatory frameworks whereby the government verifies organic certifications consolidate not only the process of harmonization by creating common standards, however they also consolidate the consumers’ trust in buying organic products. In line with this, we can encourage farmers by applying tax reductions/exemptions for organic food producers or support them in research and marketing.

According to Offermann et al. [38], it is supposed that the government play an important role in the financial viability of organic farms in both Western and Eastern European countries. In line with this, Padel et al. [39], based on analysis of EU Regulation 2078/92, EU regulation 2092/91 and using information provision of organic farming, conclude that the variation in support policies alone cannot explain the different rates of conversion to organic farming in the EU.

Regarding organic food consumers, it is found that their decisions are influenced by quality and safety of products [10] and according to a judgment developed by Nelson [40] and Darby et al. [41], the choice is more guided by credence characteristics. It is important to point out that several economic studies analysed organic products as credence goods [40,41,42,43,44] and point out the implication of quality and attributes that cannot be avoided by buyers. The scientific basis regarding organic food consumers highlights in terms of price Premiums for Organic Food a variability from 10% to as high as 50% depending on the country: Switzerland 10–20%, United Kingdom 30–50%, Sweden 20–40%, Denmark 20–30%, Austria 25–30% [45].

Having in mind that consumers may only know that the product is organic when they are informed [44], the variability of price premium for organic food may be influenced by information about these type of products, which in line with Nelson [40], Darbi [41], Hansen [43] and Giannakas’ [44] point of view, is asymmetric and obstructs the consumers view regarding organic characteristics. In other words, it is possible for organic food consumers to skip detailed information regarding organic food attributes in terms of environmental concerns, animal welfare and food safety (health concerns), having in mind only the idea of chemical avoidance. So, the consumers may only know that the product is organic when they are informed in terms of organic ingredients, organic certified and there being an organic seal.

As far as the combination of traditional knowledge, scientific evidence, climate change preoccupations, policy framework and regulation putting pressure on and deepening the security issues for the area of organic food, it is necessary for the contemporary period to come up with solutions for consolidation, not only for the policy makers’ decisions in terms of harmonization of legislation and business plans, however also to keep the goals of sustainability. Even if we find the international standards for organic agriculture (Codex Alimentarius and International Federation of Organic Agriculture Movements-IFOAM), certification requirements and regulation should not be an obstacle to the continuation of the organic sector. To solve this problem, it requires the consolidation of a dialogue structure (law harmonization in organic agriculture) that is capable to facilitate the international trade and development of international markets.

In order to move forward towards a coherent and common policy and to offer consumers the same quality guarantees all over Europe, it is useful to analyze the subject of organic food from both legal and policy framework, highlighting the trends and challenges from this area.

## 2. Materials and Methods

Since one of our objectives was to approach the literature that addresses the topics of organic production and major challenges and vulnerabilities from this area, we have done our analyses by identifying the key definitions, approaches, advantages and disadvantages of each of the topics. We started with qualitative sequential methodology, involving empiric analysis that will provide coherence and viability for our study. More specifically, we conducted the analysis by following three stages:

**Stage I.** Content analysis establishes the state of affairs: a content analysis of relevant documents from the framework of organic food in general and the legal framework in particular. Building a concrete image of the context of organic food. Retrospective critical analysis of the European legal framework specificity by reference to the global stage for better research accuracy.

**Stage II**. Empirical Analysis of European Organic food for the period 2000–2015. Correlation of data obtained in the first stage by analysing policy implication, following the overarching strategy of the EU, within some action programs.

**Stage III.** Establishing the status of convergence to food security. Corelate the Trends in European organic food with major challenges and vulnerabilities in this area. Relate food security in terms of environment protection, healthy life, quality in food production and sustainable agriculture.

Considering the purpose of the study, the technique used is represented by the study of the documents which are mainly two categories: the reports on organic agriculture and organic regulation standards. Conceptual content analysis is followed by empiric analysis.

The review summarizes some of the key points of view and results found in the literature, presenting the retrospective of EU organic food from the point of regulation, policy framework, trends and challenges. In order to present our readers with a larger view on the subject, the papers from the literature that were used for our study were found in international databases and well-known journals and in order to give a better understanding of the subject, we explored the datasets presented by the Research Institute of Organic Agriculture (FiBL) and IFOAM [46,47].

## 3. Retrospective Analysis Regarding EU Organic Regulation

Even if organic agriculture became visible on a wider scale in the 1960s, when the global sector agenda discussed the implication of chemicals used in crop and animal production by correlating them with human health and environment protection, the separation between organic and conventional food was made inherently in the context of the constitution of public or private certification bodies capable of being linked to the application of organic food standards. Hence, the organic movement in terms of the legal framework has gained a place in the spotlight of the mainstream academic researchers precisely because the new approved rules come with changes, however above all, because they identify possible vulnerabilities in this area [48]. Starting in 1997, Amaditz [49] analyzed The Organic Foods Production Act of 1990 and highlight the nightmare regarding shopping for organic food from the point of view of consumers’ difficulty in distinguishing organic products behind the states “Organically grown”. Increasing the need for a coherent picture of the nature of organic foods, the subject has started to be analyzed from both the consumer’s [50,51,52,53,54,55] and the producer’s point of view [56,57,58,59,60].

With reference to the global stage, the literature indicates that by 1990, there were 22 countries that adopted rules on organic food: three states opted to consolidate their own certification programs [61], four states opted to contract with an independent certification organization, which at that moment were named (Organic Certification, Organic Labeling and Certification, Standard of Identity for Organic Foods, Organic Farm Advisory Board [62]) and 15 countries defined organic food and production techniques however did not provide any government oversight of certification (see, for example, the case of Alaska- Sale of Organic Foods or California Organic Food Act [61]). At that moment, certification was not mandatory and organic producers decided to adopt organic food statute however with consistent differences throughout the nation [62]. Thus, given that nonlinearity in regulation has caused negative implications both for consumers who were skeptical about the consumption of organic products as well as for producers who suffered from a decrease in sales, in order to eliminate guesswork and behavior of many consumers to shy away from buying organic because of confusing labels, starting from 1991, the organic food industry continued to struggle in its efforts to self-regulate and develop a consensus across the states.

On the EU stage, the retrospective image emphasizes a continued development of the organic sector and the legal framework continues to change and consolidate a stronger background for the organic sector. The requirements in this area are set by the Council Regulation (EC). As can be seen in Table 1, in 2007, we find the Council Regulation (EC) No. 834/2007, which came into force as of 1 January 2009 and sets a certain route in terms of purpose, objectives and principles of organic farming and production. Specifically, the new regulation from 2009 aims to promote sustainable development of organic agriculture by consolidating the effective functioning of the internal market and guaranteeing fair competition.

Later, by implementing two regulations: the regulation (EC) No. 889/2008 of 5 September, 2008 and the regulation (EC) No. 1235/2008 of 8 December, 2008 this specifically details the organic production, labelling, control and import rules. Article 249 of the Treaty establishing the European Community, OJ (2006) C321E also consolidates the force of law within all Member States of the European Union, however this does not mean that the regulatory divergences are missing, and the legal framework is handled in an identical manner in all EU member states. So, every country can decide to consolidate the organic legal framework at the national or regional level, following of course the structure of the Codex Alimentarius Guidelines.

These changes in the organic food legal framework indicate that as far as the idea of organic food continuing to develop, the implications of the legal framework have been called to become more and more solid and the European Union has been making imprints and working towards updating the rules concerning organic yeast (No. 1254/2008), organic aquaculture (No. 710/2009) and organic wine (No. 203/2012). Council Decision (EU) 2017/2307 of 9 October, 2017 approves the Organic Trade Agreements and have implications for the conclusion of the Agreement between the European Union and the Republic of Chile on trade of organic products. Based on this agreement, the Union and Chile accept imports into their territory and the introduction of the market of organic products.

The review on organic regulation highlights that the European Parliament tried to provide consumers with “certainty” over the quality of organic products and to consolidate a harmonized approach across the EU countries. Most of the rules carry out to offer consumers the same quality guarantees all over Europe and to consolidate the controls in order to ensure consumer confidence in the sector of organic food. From another point of view, all the changes regarding the legal framework of organic food could be a signal that food safety is lacking and that there is a need to deepen research in order to support consumer confidence in the growing organic food market [63]. Starting from 1999, the European Council Regulation was quite strict and the farm area for production, Slaughter Age, treatment used (Allopathy or Antibiotics) also encounters restrictions. So far, the status of regulation supports the organic food market power which requires the elimination of the issues of consumers and producers regarding ecologically grown products labels, certifications or quality. In order to take advantage of this booming market (in terms of assuring environment protection and healthy life), it is important to create a solid legal framework that is capable of putting pressure not only on policy makers decisions in this line, however also to eliminate the issues from the point of view of consumers and producers. When we refer to a better legal framework, we recommended that the regulatory area must be addressed: the role of both the state and private sector in consolidation standards and rules capable to develop this sector, the harmonization of national standards with CODEC and IFOAM.

## 4. Trends and Challenges Regarding European Organic Food

Based on concerns about food consumption and human health, as well as the environmental consequences that entail, organic food availability has increased at the same time with the desire to improve vertical cooperation between science, research, policy makers’ decisions and farmers’ strategy, consolidating a synergy between Member States [64,65,66,67,68]. From the point of view of policy implication, following the overarching strategy of the EU, within some action programs we identified the desire to make the European Union most competitive. Starting with the Lisbon strategy initiated by the head of states, with the first document which indicates a new focus at the 2005 Spring Summit, it can be clear that the main objective of organic food is to create great opportunities for economic growth and stability. Other institutional construction comes with change through the Common Agricultural Policy which is considered the most highly resistant to change [69,70,71,72] precisely because it sets the types of agents participating in the formulation of the policy and establishes the political processes guiding the field [73]. Institutional developments linking organic farming and the Common Agricultural Policy (CAP) are delimited in three periods. The first period runs from 1980 to 1985 and focuses on environmental concerns among all of the central agents within the CAP. The second one starts during 1986 and ends in 1992 and is based on solving the issues regarding budget pressure, distribution of public support among farmers and regions or agricultural surplus production. The last period (from 1993 to 2003) focuses on rural development problems, food safety and security and trying to clearly solve the conflicts over the boundaries of what distinguishes organic farming from other concerns, about the sorts of processes that should guide a field concerned with organic farming and about which agents should be included in and excluded from a field concerned with organic farming [74]. However, even if future scenarios indicate at that moment that some of the problems linking intensive agricultural production and environment sustainability are solved by the fact that they were translated and institutionalized within the Common Agricultural Policy (CAP), the sustainable management of organic food production and consumption still creates concerns in terms of surplus production, safety and quality. Since Common Agricultural Policy (CAP) remains a key policy for guiding the agriculture in Europe, under Pillar 1 (responsible for certified organic farmers qualifying for the “greening” payments) and Pillar 2 (which is responsible for Rural Development Programmes, RDPs), recent literature analyses the implication of the last pillar and concludes that CAP still disproportionally favors production, instead assessing the overall sustainability of the farm [75].

CORE Organic (in fact, a special European fund) is another key point project in the institutional construction of the EU which aims to improve the coordination of transnational research in organic food and farming. Launched in 2016 as part of the European Commission’s ERA-NET Scheme, the project consolidates coordination and collaboration based on 13 public funding bodies representing 11 countries: Austria, Denmark, Finland, France, Italy, Germany, Sweden, Switzerland, Netherlands, Norway and the UK. The main objective of ERA-NET (network of European ministries and research councils funding research in organic food systems at national levels) is to coordinate research and highlight the most important challenges along the organic value chains. The results of cooperation in CORE organic ERA-net are related in eight pilot research projects and imply: Methods to improve quality for organic wheat; planning for better animal health and welfare; strategies to communicate ethical values; a tool to prevent diseases and parasites in organic pig herds; more organic food for young people; methods for assessing and reducing risks of pathogen contamination; methods to make organic milk healthy; strategies to assure safety, health and sensory qualities of organic products.

As a continuity for ERA-NET action, CORE Organic Plus come with CORE organic I and CORE organic II. The last one, CORE organic II, implies 26 partners from 21 countries/regions and benefits from an additional top funding by the European Commission. Analyzing the projects that have been carried out so far, we can find both positive and negative implications, the nature of the research cooperation, as well as the gaps generated by the research topics (for example, according to Organic CORE Platform, on Finland’s profile, it has been identified that the subject of farm economics gained 0.25% of total projects). More than that, it will be useful if some research areas are grouped into clusters according to the geographical region because even if many projects are carried out on a certain subject area in each country, this necessarily results in an overlapping, being clear that the results are influenced by biological, social and political situations, which are different in EU countries.

The preoccupation for food production to minimize environmental impact comes with practices that can increase the costs of production. In order to facilitate the practices in this area, Technology Platform (TP) Organics is the European Technology Platform for Organic Food and Farming which helps strengthen research and innovation for organics and other agroecological approaches. Based on TP Organics’ implication, the idea that organic farming is an innovative approach to agriculture which can help solve the challenges and vulnerabilities of European food has been consolidated. A problem of TP Organics’ projects consists of the fact that even if in the operational group of the Innovation Arena, we find a lot of listed countries. Usually in the TP Organics’ brochure, we find a few active countries, which means that member states should stimulate exchange between countries.

**Figure 1:**
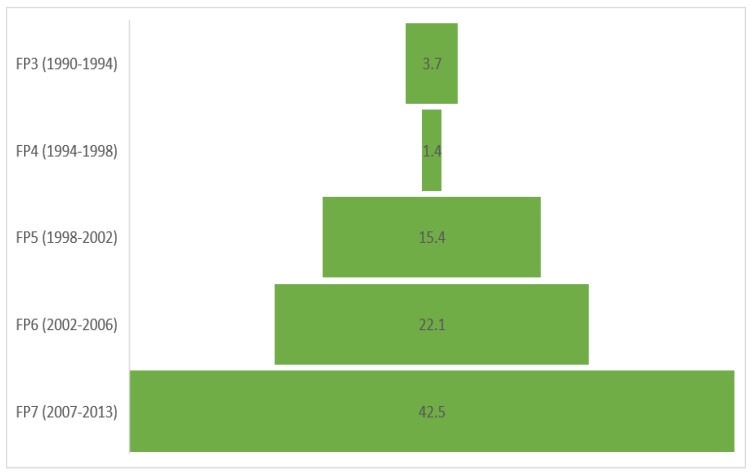
Funding of organic farming research in the EUs framework programs’ FP (million euros). Source: CORDIS database, https://cordis.europa.eu/project/rcn/111296_en.html [77].

The huge potential of organic food production and consumption is related not only by institutional implication and market trends in this area, however also by orientation to funding organic farming research. As seen in Figure 1, the trend is increasing over the years. If between 1990 and 1994 we have 3.7 million euros, in 2007–2013, the sums allocated exceed 40 million euros. According to Niggli et al. [76], we find that despite the total spending on research, the gap of the domain still exists by the fact that the research agenda does not adequately reflect correct dissemination. The author highlights that many scientific findings are still poorly developed and many practical problems are not yet resolved. Also, in order to assure the basis for scientific progress, it is necessary to argue the opinions of some critics of organic agriculture. Based on this, we can come to the conclusion that the problems occurred from the point that the research agenda should be capable of identifying the major challenges of the next years and to be proactive from the point of policy implication and food security.

An adequate development in terms of organic food should be corelated with the demand for processing this type of product by analysing the trend of crops grown. As can be seen in Table 2, Europe have experienced a rapid growth in the arable crops group, the largest being cereals, which recorded 2.2 million hectares and on the same trend, 1.7 million hectares in the EU. Based on FiBL AMI survey 2016, the largest cereals area is found in Germany, Spain and Italy and outside the EU, Ukraine, Russia and Turkey are major producers [78]. In terms of organic vegetables, Table 2 shows that they are grown on over 157,000 hectares of land in Europe and more than 140,000 hectares in the EU and linked to a recent research on this subject [65], the trends regarding development of land related to organic vegetables will continue to grow in the next year.

**Figure 2:**
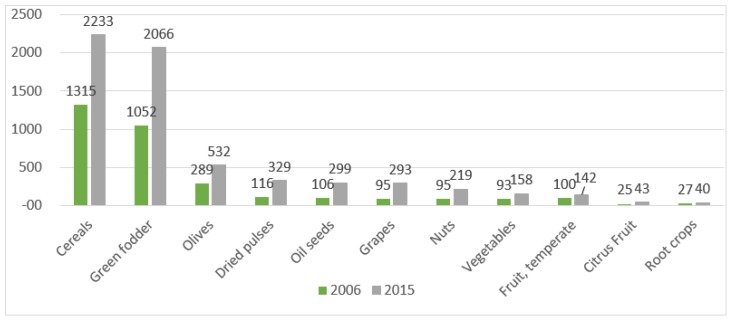
Development of selected crop groups in Europe 2006–2015 (thousand hectares). Source: Source: FiBL-AMI survey 2016.

The high demand of the European organic market is shown by the trend recorded on the profile of oilseeds with +22 percent from 2014 to 2015, vegetables with +19 percent and cereals with +17 percent. The period from 2006 to 2015 recorded the highest growth for dry pulses and oilseeds (+180 percent each). Across Europe, we find high growth rates between 2006 and 2015 for the development of grapes +208 percent and citrus +70 percent (Table 2). These results are also correlated to the trend related in Figure 2 which reflects the higher development on the profile of cereals (1315 hectares in 2006 and 2233 hectares in 2015) and green folder, with 1052 hectares in 2006 and 2066 in 2015. A common view among the general picture allows us to assume the following judgement which is also linked to the literature on this subject—because the food demand still requires major challenges in agricultural production systems, a major vulnerability of organic food production consists of the capability to improve cropland management without degrading soil and water resources [79].

**Figure 3:**
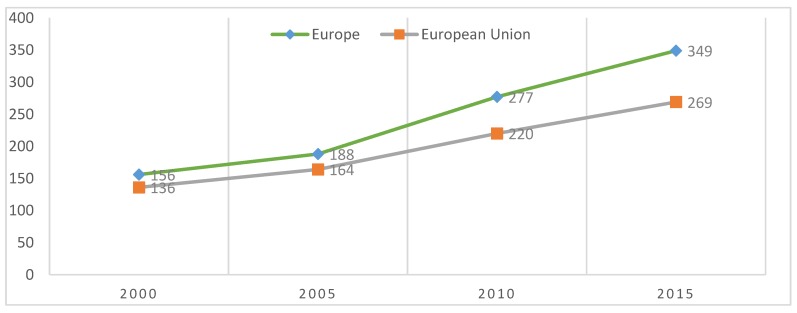
Development of organic producers 2000–2015 (in thousands of producers). Source: FiBL-AMI survey 2006–2017 based on national data sources and Eurostat.

Producers processors, importers and exporters are important actors in the development of organic food. Based on processors as well as producer’s capability to be focused on food quality and food system issues, consumers’ behavior to purchase organic food can be identified and the potentials of organic agriculture can be explored [58,80]. Importers and exporters are actors that influence trade in organic food and guide international demand [57]. Regarding organic producers, it is highlighted at the level of year 2015 that there are almost 350,000 organic producers in Europe and almost 270,000 in the EU. Changes from 2006 to 2015 recorded a huge growth from the point of producers, specifically 71% for total Europe (Table 3). Since 2000 to 2015, the development of organic producers follows a positive trend, increasing from 156,000 producers in 2000 to 349,000 (case of Europe) and from 136,000 producers to 269,000 (in the case of the European Union)—Figure 3. The growing number of producers, over 70% from 2006 to 2014, reflects from one side the importance of organic agriculture in human health (as far as organic food consumption, it may reduce the risk of allergic disease and of being overweight and obesity) and from another side, the assumption that EU countries should be championed for efforts to strengthen consumer confidence in organic products. The last point can be fulfilled by assuring food safety, environmental sustainability and achieving consistent standards capable of positively impacting farmers’ development. The producers’ trend is also influenced by consumer preferences and attitudes towards organically grown produce, and according to the literature, they become more and more interested in the field [81,82,83,84,85]. Organic producers and importers increased in almost all European countries and in the EU, there were almost 60,000 processors (over 60,000 in Europe), almost 3500 importers in the EU and 3681 importers in Europe (Table 3).

**Figure 4:**
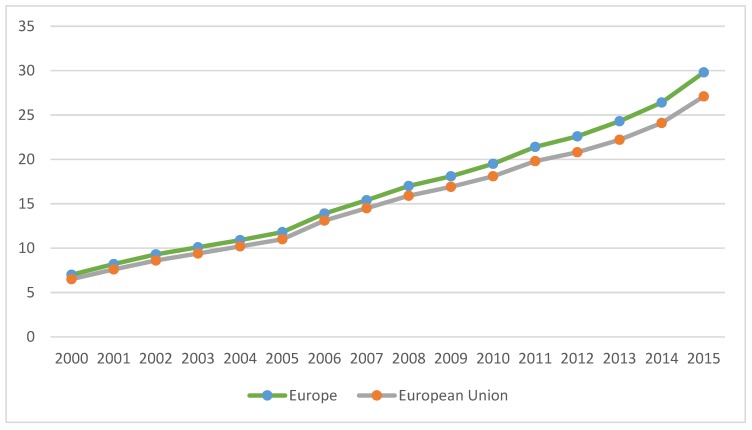
Growth of organic retail sales in Europe and the European Union, 2000–2015 (Billion euros). Source: FiBL-AMI Surveys 2006–2017 and Organic Data Network Surveys 2013–2015.

**Figure 5:**
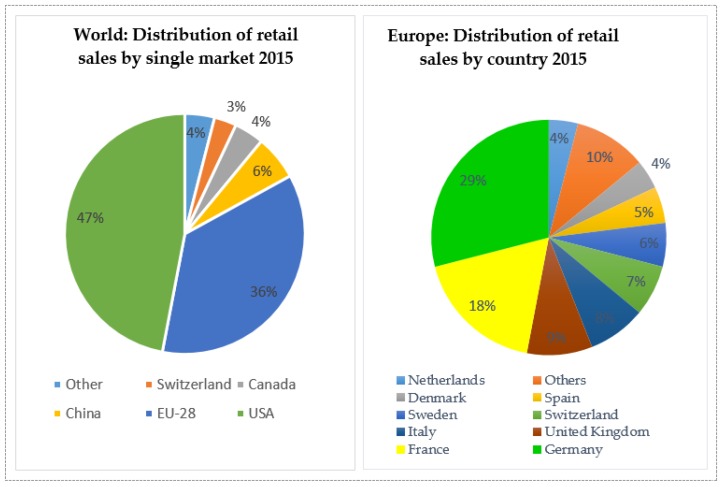
Distribution of retail sales by country and by single market worldwide. Source: FiBL-AMI survey 2017 based on national data sources.

Despite the financial crisis that occurred in 2008, the organic market in Europe and the European Union grew by approximately double, from 17 billion euros in 2008 to almost 30 in 2015 and from 15.9 billion euros in 2008 to 27.1 in 2015, on the profile of the European Union (Figure 4). Comparing the organic market to the single market, USA has the lead, with 47% of global retail sales of organic products (almost 36 billion euros), followed by the European Union, with 35 percent of global retail sales (almost 27 billion euros)—Figure 5.

**Figure 6:**
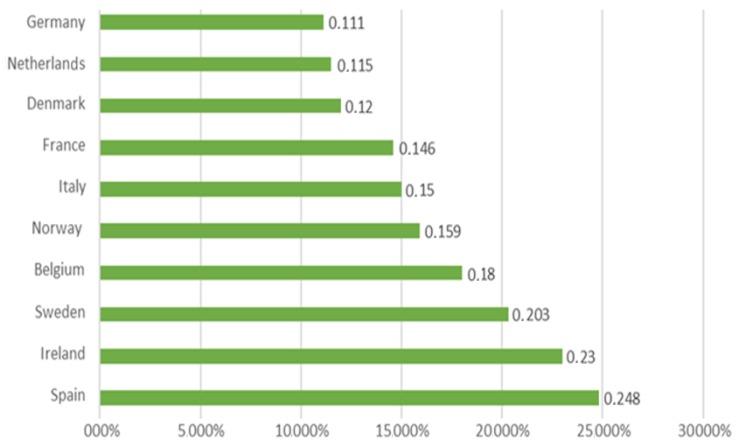
The countries with the highest growth of the organic market 2015 (market growth in %). Source: FiBL-AMI Surveys 2017.

In 2015, the top three countries with the highest growth of the organic market are Spain with 24.8%, Ireland with 23.0% and Sweden with 20.3%; the lowest results are on the profile of the Netherlands and Germany, with 11.5% and 11. 1%, respectively (Figure 6).

**Figure 7:**
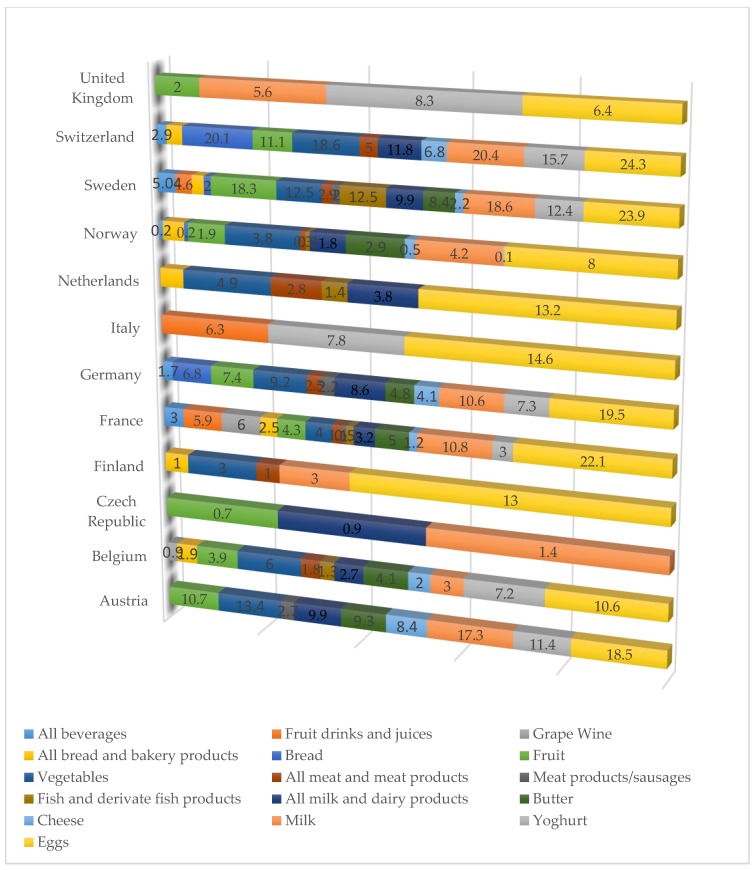
Shares of organic products and product groups of their respective total markets for selected countries in 2015. Source: Austria: AMA Marketing, Belgium, LF based on GFK, Czech Republic: UZEI and experts estimates, Finland: Pro Luomu, France: Agence BIO; Germany: Agricultural market information Company based on GFK; Italy: Asso Bio based on Nielsen; Netherlands: Bionext; Norway: Nielsen Norway; Sweden: Statistics Sweden; Switerland: Bio Suisse; UK: Soil Association.

In many countries, organic eggs recorded the highest values within the total retail market, with France, Switzerland and Sweden having recorded values over 20 percent. In line with this, the literature highlights that consumers are willing to pay a premium price for organic eggs instead of cage produced eggs [86]. Other popular products are represented by organic fruit and vegetables, organic vegetables being in second place after eggs, with the highest market share on the profile for Germany, Sweden, Austria and Switzerland (Figure 7).

It is important to notice that the Czech Republic and France data is from 2014 because of the availability. On the profile of France, all beverages include vegetable drinks, fruit and vegetable juices, wine and alcohol. On the profile of Sweden, all beverages exclude alcoholic beverages. On the Norway profile, all bread and bakery products include groats, flower, bread, crisp bread, pasta, rice, breakfast cereals and other cereal products. In Switzerland, all bread and bakery products include bread and bakery products other than fresh bread. Regarding vegetables, in Finland, it includes fruits and in the Netherlands, it include vegetables and fruit. All meat and meat products in Switzerland include fish and fish products. All milk and dairy products exclude eggs in Sweden.

## 5. Conclusions

This study aims to analyze and empirically evaluate the EU organic food from the point of view of regulation, policy framework, trends and challenges. Based on a critical review of existing scientific evidence regarding European trends in organic food consumption and production, we identified research gaps for future comprehensive assessments of the policy and legal framework and we highlighted the importance of the two pillars, namely regulation and policy. In addition, the paper examines the need for solid restrictions regarding organic food production, however also the need to support food safety and consumer confidence in the growing organic food market.

Our conceptual content analysis, followed by empiric analysis, indicates that EU countries today are concerned with security issues raised by growth in the organic food market. The creation of a solid framework requires the food to be labeled as “environmentally-friendly” or “green” and to illustrate how the consumption of this type of food keeps improving humans’ health by avoiding the risk of allergic diseases, of being overweight and obesity, however also to improve the environment protection. It is not only necessary that the farmers consolidate an efficient management, however it is also necessary that they are supported by adequate policy and regulation framework. If the future legal framework takes into consideration not only the organic standards and certifications, however also the consolidation of the process of harmonization by creating common standards, which can develop the consumer’s trust in buying organic products, then the final output will consist of the development of organic food. Also, it is required that governments are linked to producer’s needs, however also to include in the long-term strategy for local development the encouragement of farmers by applying tax reductions/exemptions for organic food producers or to support them in research and marketing.

With reference to the retrospective view regarding EU organic regulations, all the changes in the legal framework of organic food could be a signal that food safety is lacking and that there is a need to deepen research in order to support consumer confidence in the growing organic food market. In line with this, institutional implication can guide market trends in this area, developing the orientation to fund organic farming research. Our results show that despite the total spending on research, the gap of the domain still exists by the fact that the research agenda does not adequately reflect correct dissemination. More specifically, many scientific findings are still poorly developed and many practical problems are not yet resolved. Also, in order to assure the basis for scientific progress, it is necessary to argue the opinions of some critics of organic agriculture. The findings of the study reveal that the problems occurred from the point of view that the research agenda should be capable of identifying the major challenges of the next years and to be proactive from the point of policy implication and food security.

Overall, we found that the greatest challenge for organic food is to reduce the trade-offs between productivity and long-term sustainability. Even if the EU countries have their own particularities in terms of the status of organic food, the inappropriate design of policy and legal framework can obstruct accession of EU organic food and require coordination, harmonization and common mechanisms and standards that are capable of supporting organic producers and to avoid the issues related to organic food development and environmental protection.

## Figures and Tables

**Table 1:** EU organic regulations history.

Year	Law	Aim and Implications
2018	Regulation (EU) 2018/848 of 30 May, 2018 on organic production and labelling of organic products	Repeal the Council Regulation (EC) No 834/2007 and given the fact that this new rule will apply from 1 January, 2021, many details from the body of law that will be developed in future in this moment has been adopted as the “Basic Act”. The law will contain production rules for farmers, production rules for food processors and new requirements in terms of Control and certification.
2017	Council Decision (EU) 2017/2307 of 9 October, 2017	Consolidation of the Organic Trade Agreements and have implications on the conclusion of the Agreement between the European Union and the Republic of Chile on the trade of organic products.
2008	Regulation (EC) No 889/2008 of 5 September, 2008	Laying down detailed rules for the implementation of Council Regulation (EC) No 834/2007 on organic production and labelling of organic products with regard to organic production, labelling and control.
Council Regulation (EC) No 834/ of 28 June, 2007 was Amended by:	Regulation (EC) No 1254/2008 on organic yeast, use of in-conversion feeding stuffs, decorative colouring of egg shells, use of enzymes; Regulation (EC) No 710/2009 detailing rules for organic aquaculture and seaweed production; Regulation (EU) No 271/2010 on the new organic logo; Regulation (EU) No 344/2011 on the clarification on labelling and on the use of rosemary extract; Regulation (EU) No 426/2011 on the publication of information; Regulation (EU) No 126/2012 on the equivalency agreement with the USA; Regulation (EU) No 203/2012 detailing rules on organic wine production; Regulation (EU) No 505/2012 on feed and correcting provision on the use of extracts of rosemary; Regulation (EU) No 392/2013 amending the control system.	Regulation (EU) No 519/2013 adaptation to EU enlargement to Croatia; Regulation (EU) No 1030/2013 postponing a deadline for organic aquaculture; Regulation (EU) No 1364/2013 on aquaculture non-organic juveniles and seed; Regulation (EU) No 354/2014 correcting a mistake and amending annexes I, II, V and VI; Regulation (EU) No 836/2014 postponing deadlines for using non-organic young poultry and protein feed; Regulation (EU) No 1358/2014 on aquaculture; Regulation (EU) 2016/673 on aquaculture, wine, Annexes II, VI & VIII; Regulation (EU) 2017/838 on aquaculture feed Regulation (EU) 2017/2273 postponing deadlines for using non-organic young poultry and protein feed; Regulation (EU) 2018/1584 on beekeeping, shrimps and prawns, young pullets, protein feed, babyfood, information exchange and Annexes I, II and VIIIa.
2008	Regulation (EC) No 1235/2008 of 8 December, 2008	Laying down detailed rules for implementation of Council Regulation (EC) No 834/2007 regarding the arrangements for imports of organic products from third countries.
The regulation (EC) No 1235/2008 of 8 December, 2008 was amended by:	Regulation (EC) No 537/2009 Regulation (EU) No 471/2010 Regulation (EU) No 590/2011 Regulation (EU) No 1084/2011; Regulation (EU) No 1267/2011; Regulation (EU) No 126/2012; Regulation (EU) No 508/2012; Regulation (EU) No 751/2012; Regulation (EU) No 125/2013; Regulation (EU) No 519/2013; Regulation (EU) No 567/2013; Regulation (EU) No 586/2013; Regulation (EU) No 355/2014; Regulation (EU) No 442/2014; Regulation (EU) No 644/2014.	Regulation (EU) No 1287/2014 Regulation (EU) 2015/131 Regulation (EU) 2015/931 Regulation (EU) 2015/1980; Regulation (EU) 2015/2345; Regulation (EU) 2016/459; Regulation (EU) 2016/910; Regulation (EU) 2016/1330; Regulation (EU) 2016/1842; Regulation (EU) 2016/2259; Regulation (EU) 2017/872; Regulation (EU) 2017/1473; Regulation (EU) 2017/1862; Regulation (EU) 2017/2329; Regulation (EU) 2018/949.
2007	Council Regulation (EC) No 834/ of 28 June, 2007 defining the aims, objectives and principles of organic farming and production, and by implementing two regulations (No 889/2008 and No 1235/2008) detailing the organic production, labelling, control and import rules. All products labelled as organic and sold in the EU must be produced in accordance with these regulations.	Set the principles, aims and overarching rules of organic production and defining how organic products were to be labelled. This law repealed Regulation (EEC) No 2092/91 and was amended by: Regulation (EC) No 967/2008 postponing the obligation to use the organic logo Regulation (EU) No 517/2013 adaptation to EU enlargement to Croatia. Regarding the old EU Regulation 2092/91, it is found that it influenced the expansion of the Codex Alimentarius Guidelines in the late 1990s.

**Table 2:** Key crops and crop group in Europe and the European Union (2015).

Land Use	Crop Group	Europe (ha)	EU (ha)	Organic Share of Total (%) EU	Change 2014–2015 (%) EU	Change 2006–2015 (%) EU
Arable crops	Cereals	2,232,921	1,681,274	1.7% (2.9%)	17% (10%)	70% (49%)
	Dry pulses	328,870	299,930	6.7% (21.2%)	10% (18%)	183% (195%)
	Green folder	2,065,761	1,867,966	9.6% (11.2%)	1% (2%)	96% (93%)
	Oilseeds	298,856	210,855	1% (1.8)	22% (25%)	182% (137%)
	Root crops	40,105	30,726	0.4% (0.9%)	−4% (−0.5%)	47% (34%)
	Vegetables	157,964	143,012	3.1% (6.2%)	19% (20%)	69% (61%)
Arable crops total		5,661,759	4,738,463	2.8% (5.0%)	5% (7%)	87% (74%)
Permanent crops	Berries	31,638	29,767	11.2% (17.7%)	1% (4%)	458% (436%)
	Citrus fruit	42,520	42,166	6.3% (7.7%)	9% (9%)	70% (73%)
	Fruit temperate	141,517	120,161	4.7% (8.7%)	11% (11%)	41% (44%)
	Fruit, (sub)tropical	26,455	9196	7.3 (3.7%)	−16% (9%)	3252% (1089%)
	Grapes	292,753	281,139	7.3% 8.8%)	10% (12%)	208% (231%)
	Nuts	219,164	189,704	13.2% (20.3%)	21% (23%)	131% (105%)
	Olives	532,083	453,152	9.1% (9.1%)	8% (9%)	84% (62%)
Permanent crops total		1,397,140	1,229,390	8.7% (10.6%)	3% (3%)	102% (105%)
Total cropland		7,058,898	5,967,854	3.2% (5.6%)	5% (7%)	89% (79%)

Source: FiBL-AMI survey 2016 based on national data sources and Eurostat (1,2).

**Table 3:** Organic operators by country.

Producers				Processors		Importers		Exporters	
Country Group	No.	Change 2014–2015	Change 2006–2015	No.	Change 2014–2015	No.	Change 2014–2015	No.	Change 2014–2015
EU-28	269,453	5%	50%	58,360	12%	3474	19%	1957	124%
EU-15	207,425	7%	34%	55,722	12%	3135	19%	1759	144%9
EU-13	62,028	−1%	143%	2638	14%	339	28%	198	32%
CPC	71,033	−2%	381%	1160	23%	79	20%	88	6%
EFTA	8431	−1%	−6%	387	-25%	77	15%	4	0%
Other European countries	344	−19%	65%	166	66%	51	−15%	51	−11%
Total Europe	349,261	3%	71%	60,073	12%	3681	19%	2100	107%

Source: FiBL-AMI survey 2016 based on national data sources and Eurostat (1,2).

## References

[1] Bourn D., Prescott J. (2002). A Comparison of the Nutritional Value, Sensory Qualities, and Food Safety of Organically and Conventionally Produced Foods. Crit. Rev. Food Sci. Nutr..

[2] Klonsky K., Tourte L. (1998). Organic Agricultural Production in the United States: Debates and Directions. Am. J. Agric. Econ..

[3] Goldman M.C., Hylton W. (1972). The Basic Book of Organically Grown Foods.

[4] Magkos F., Arvaniti F., Zampelas A. (2003). Organic food: nutritious food or food for thought? A review of the evidence. Int. J. Food Sci. Nutr..

[5] Scialabba N., Hattam C. (2002). Organic Agriculture, Environment and Food Security.

[6] Snyder C., Spaner D. (2010). The Sustainability of Organic Grain Production on the Canadian Prairies—A Review. Sustainability.

[7] Gomiero T. (2013). Alternative Land Management Strategies and Their Impact on Soil Conservation. Agriculture.

[8] Torjussen H., Nyberg A., Wandel M. (1999). Organic Food; Consumers’ Perceptions and Dietary Choices. Statens Inst. for Forbruksforskning, Report No. 5. www.sifo.no/english/publications/environment.

[9] Mukherjee A., Lal R. (2013). Biochar Impacts on Soil Physical Properties and Greenhouse Gas Emissions. Agronomy.

[10] Vindigni G., Janssen M.A., Jager W. (2002). Organic food consumption: A multi-theoretical framework of consumer decision making. Br. Food J..

[11] Velten S., Leventon J., Jager N., Newig J. (2015). What Is Sustainable Agriculture? A Systematic Review. Sustainability.

[12] Hartman G.L., Pawlowski M.L., Herman T.K., Eastburn D. (2016). Organically Grown Soybean Production in the USA: Constraints and Management of Pathogens and Insect Pests. Agronomy.

[13] Montanarella L., Lugato E. (2013). The Application of Biochar in the EU: Challenges and Opportunities. Agronomy.

[14] Hati K.M., Swarup A., Dwivedi A., Misra A., Bandyopadhyay K. (2007). Changes in soil physical properties and organic carbon status at the topsoil horizon of a vertisol of central India after 28 years of continuous cropping, fertilization and manuring. Agric. Ecosyst..

[15] Kundu S., Singh M., Saha J.K., Biswas A., Tripathi A.K., Acharya C.L. (2001). Relationship between C addition and storage in a Vertisol under soybean-wheat cropping system in sub-tropical central India. J. Plant Nutr. Soil Sci..

[16] Manna M., Swarup A., Wanjari R., Ravankar H., Mishra B., Saha M., Singh Y., Sahi D., Sarap P. (2005). Long-term effect of fertilizer and manure application on soil organic carbon storage, soil quality and yield sustainability under sub-humid and semi-arid tropical India. Field Crops Res..

[17] Reeder J., Schuman G., Bowman R. (1998). Soil C and N changes on conservation reserve program lands in the Central Great Plains. Soil Tillage Res..

[18] Schuman G. (2002). Soil carbon dynamics and potential carbon sequestration by rangelands. Environ. Pollut..

[19] Bhattacharyya R., Kundu S., Prakash V., Gupta H. (2008). Sustainability under combined application of mineral and organic fertilizers in a rainfed soybean–wheat system of the Indian Himalayas. Eur. J. Agron..

[20] Schulz H., Glaser B. (2012). Effects of biochar compared to organic and inorganic fertilizers on soil quality and plant growth in a greenhouse experiment. J. Plant Nutr. Soil Sci..

[21] Nkoa R. (2014). Agricultural benefits and environmental risks of soil fertilization with anaerobic digestates: A review. Agron. Sustain. Dev..

[22] Lee D.R. (2005). Agricultural Sustainability and Technology Adoption: Issues and Policies for Developing Countries. Am. J. Agric. Econ..

[23] Senesil G.S., Baldassarre G., Senesi N., Radina B., Senesi G. (1999). Trace element inputs into soils by anthropogenic activities and implications for human health. Chemosphere.

[24] Horrigan L., Lawrence R.S., Walker P. (2002). How sustainable agriculture can address the environmental and human health harms of industrial agriculture. Environ. Health Perspect..

[25] Worthington V. (2001). Nutritional Quality of Organic Versus Conventional Fruits, Vegetables, and Grains. J. Altern. Complement. Med..

[26] Pimentel D., Burgess M. (2013). Soil Erosion Threatens Food Production. Agriculture.

[27] Damalas C.A., Eleftherohorinos I.G. (2011). Pesticide Exposure, Safety Issues, and Risk Assessment Indicators. Int. J. Environ. Res. Public Health.

[28] Harper G.C., Makatouni A. (2002). Consumer perception of organic food production and farm animal welfare. Br. Food J..

[29] Hughes D. (1995). Animal welfare: The consumer and the food industry. Br. Food J..

[30] Baudry J., Méjean C., Allès B., Péneau S., Touvier M., Hercberg S., Lairon D., Galán P., Kesse-Guyot E. (2015). Contribution of Organic Food to the Diet in a Large Sample of French Adults (the NutriNet-Santé Cohort Study). Nutrients.

[31] European Section of the Organic World. https://www.organic-europe.net/europe-statistics/europe-statistics-news.html.

[32] FAOSTAT FAO (Food and Agricultural Organization) Statistical Data. http://faostat3.fao.org/home/E.

[33] Crush J., Bruce F. (2014). Feeding African cities: The growing challenge of urban food insecurity. Africa’s Urban Revolution.

[34] Murage A.W., Midega C.A.O., Pittchar J.O., Pickett J.A., Khan Z.R. (2015). Determinants of adoption of climate-smart push-pull technology for enhanced food security through integrated pest management in eastern Africa. Food Secur..

[35] Lal R. (2004). Soil Carbon Sequestration Impacts on Global Climate Change and Food Security. Science.

[36] Frison E.A., Cherfas J., Hodgkin T. (2011). Agricultural Biodiversity Is Essential for a Sustainable Improvement in Food and Nutrition Security. Sustainability.

[37] Ceccarelli S. (2014). GM Crops, Organic Agriculture and Breeding for Sustainability. Sustainability.

[38] Offermann F., Nieberg H., Zander K. (2009). Dependency of organic farms on direct payments in selected EU member states: Today and tomorrow. Food Policy.

[39] Padel S., Lampkin N., Foster C., Lampkin N. (1999). Influence of policy support on the development of organic farming in the European Union. Int. Plan. Stud..

[40] Nelson Ph. (1970). Information and consumer behaviour. J. Political Econ..

[41] Darby M.R., Karni E. (1973). Free Competition and the Optimal Amount of Fraud. J. Law Econ..

[42] Andersen E.S., Philipsen K. (1998). The Evolution of Credence Goods in Customer Markets: Exchanging “Pigs in Pokes”.

[43] Hansen L.G. Modeling Demand for Organic Products—Implications for the Questionnaire. Working Paper #4.AKF. 2001. Danish Institute of Local Government Studies. http://www.akf.dk/organicfoods/papers/wp4-lgh.pdf.

[44] Giannakas K. (2002). Information Asymmetries and Consumption Decisions in Organic Food Product Markets. Can. J. Agric. Econ..

[45] Turco G. Organic Food-An. Opportunity, at Who’s Expense? Industry Note. Food and Agribusiness Research; Rabobank International, Sydney. http://www.rabobank.com/attachments/in-043-2002.

[46] Research Institute of Organic Agriculture (FiBL). https://statistics.fibl.org/.

[47] Statistical yearbook “The World of Organic Agriculture”, IFOAM. https://www.ifoam.bio/en/news/2017/02/09/world-organic-agriculture-2017.

[48] Gibbon P. (2008). An Analysis of Standards-based Regulation in the EU Organic Sector, 1991–2007. J. Agrar. Chang..

[49] Amaditz K.C. (1997). The Organic Foods Production Act of 1990 and its impending regulations: A big zero for organic food?. Food Drug Law J..

[50] Thompson G.D. (1998). Consumer Demand for Organic Foods: What We Know and What We Need to Know. Am. J. Agric. Econ..

[51] Schifferstein H.N., Ophuis P.A.O. (1998). Health-related determinants of organic food consumption in The Netherlands. Food Qual. Prefer..

[52] Latacz-Lohmann U., Foster C. (1997). From “niche” to “mainstream”—Strategies for marketing organic food in Germany and the UK. Br. Food J..

[53] Boccaletti S. (2000). Consumer willingness to pay for pesticide-free fresh fruit and vegetables in Italy. Int. Food Agribus. Manag..

[54] Katrin M., Hansen L.G. (2002). Willingness to Pay for Organic Foods: A Comparison between Survey Data and Panel Data from Denmark.

[55] Tanner C., Kast S.W. (2003). Promoting sustainable consumption: Determinants of green purchases by Swiss consumers. Psychol. Mark..

[56] Lohr L. (1998). Implications of Organic Certification for Market Structure and Trade. Am. J. Agric. Econ..

[57] Lohr L. (2001). Factors Affecting International Demand and Trade in Organic Food Products. Changing Structure of Global Food Consumption and Trade.

[58] Torjusen H., Lieblein G., Wandel M., A Francis C. (2001). Food system orientation and quality perception among consumers and producers of organic food in Hedmark County, Norway. Food Qual. Prefer..

[59] Morgan K., Murdoch J. (2000). Organic vs. conventional agriculture: Knowledge, power and innovation in the food chain. Geoforum.

[60] Raynolds L.T. (2000). Re-embedding global agriculture: The international organic and fair trade movements. Agric. Hum. Values.

[61] Ellsworth J. (2001). The History of Organic Food Regulation. http://nrs.harvard.edu/urn-3:HUL.InstRepos:8889458.

[62] Bones G.G. (1992). State and Federal Organic Food Certification Laws: Coming of Age? Originally Published in North Dakota Law Review, No. 405. http://nationalaglawcenter.org/wp-content/uploads/assets/bibarticles/bones_organic.pdf.

[63] Siderer Y., Maquet A., Anklam E. (2005). Need for research to support consumer confidence in the growing organic food market. Trends Food Sci. Technol..

[64] Schmid O., Dabbert St., Eichert Ch., Gonzálvez V., Lampkin N., Michelsen J., Slabe A., Stokkers R., Stolze M., Stopes Ch. (2008). Development, implementation and evaluation. A Resource Manual for the Organic Food and Farming Sector.

[65] Padel S., Jasinska A., Rippin M., Schaack D., Willer H., Willer H., Yussefi-Menzler M., Sorensen N. (2008). European Market for Organic Food in 2006. The World of Organic Agriculture—Statistics and Emerging Trends, IFOAM and FIBL.

[66] Willer H., Yussefi-Menzler M., Sorensen N., The World of Organic Agriculture (2008). Statistics and Emerging Trends 2008.

[67] Willer H. (2009). The World Organic Agriculture—Statistics and Emerging Trends.

[68] Llorens Abando L., Rohner-Thielen E. Different Organic Farming Patterns within EU-25. An Overview of the Current Situation. Statistics in Focus. Eurostat. 2007, 69. http://epp.eurostat.ec.europa.eu/cache/ity_offpub/ks-sf-07-069/en/ks-sf-07-069-en.pdf.

[69] Coleman W.D., Tangermann S. (1999). The 1992 CAP reform, the Uruguay Round and the Commission: Conceptualising linked policy games. J. Common Mark. Stud..

[70] Daugbjerg C. (1999). Reforming the CAP: Policy Networks and Broader Institutional Structures. J. Common Mark. Stud..

[71] Lenschow A., Zito A. (1998). Blurring or shifting of policy frames? Institutionalisation of the economic–environmental policy linkage in the European Community. Governance.

[72] Skogstad G. (1998). Ideas, Paradigms and Institutions: Agricultural Exceptionalism in the European Union and the United States. Governance.

[73] Lynggaard K. (2007). The institutional construction of a policy field: A discursive institutional perspective on change within the common agricultural policy. J. Eur. Public Policy.

[74] Lynggaard K. (2006). The Common Agricultural Policy and Organic Farming. An Institutional Perspective on Continuity and Change.

[75] Stolze M., Sanders J., Kasperczyk N., Madsen G. (2016). Organic Farming and the Prospects for Stimulating Public Goods under the CAP 2014–2020.

[76] Niggli U., Slabe A., Schmid O., Halberg N., Schlüter M. (2008). Vision for an Organic Food and Farming Research Agenda 2025; Organic Knowledge for the Future; Food, Fairness and Ecology. http://www.organic-research.org.

[77] Coordination of European Transnational Research in Organic Food and Farming Systems- CORDIS Database. https://cordis.europa.eu/project/rcn/111296_en.html.

[78] Eurostatdatabase. http://ec.europa.eu/eurostat/product?code=org_croparandhttps://www.fibl.org/en/themes/organic-farming-statistics.html.

[79] Branca G., McCarthy N., Lipper L., Jolejole C. (2011). Climate-smart agriculture: A synthesis of empirical evidence of food security and mitigation benefits from improved cropland management. Mitig. Clim. Change Agric. Ser..

[80] Hughner R.S., McDonagh P., Prothero A., Shultz C.J., Stanton J. (2007). Who are organic food consumers? A compilation and review of why people purchase organic food. J. Consum..

[81] Huang C.L. (1996). Consumer preferences and attitudes towards organically grown produce. Eur. Agric. Econ..

[82] Wang Q., Sun J., Parsons R. (2010). Consumer Preferences and Willingness to Pay for Locally Grown Organic Apples: Evidence from a Conjoint Study. HortScience.

[83] Hjelmar U. (2011). Consumers’ purchase of organic food products. A matter of convenience and reflexive practices. Appetite.

[84] Schleenbecker R., Hamm U. (2013). Consumers’ perception of organic product characteristics. A review. Appetite.

[85] Bostan I. An Analysis of the “bio”/”eco” Products Market, Referring to the EU and Romania. CES—Working Papers. 2016, Vol. VIII, Issue 1, 33–44. http://www.ceswp.uaic.ro/articles/CESWP2016_VIII1_BOS.pdf.

[86] Gracia A., Barreiro-Hurle J., López-Galán B. (2014). Are local and organic claims complements or substitutes? A consumer preferences study for eggs. J. Agric. Econ..

